# Tafazzin-Deficient Zebrafish Display Mitochondrial Dysfunction, Neutropenia, and Metabolic Defects Without Myopathy

**DOI:** 10.21203/rs.3.rs-5960642/v1

**Published:** 2025-04-24

**Authors:** Usua Oyarbide, Rebecca A. Anderson, Igor Radzikh, Jillian V. Kodger, Akshay S. Patil, Morgan Staton, Anny Mulya, Genevieve M. Crane, Silvio Litovsky, Yana Sandlers, Seth J. Corey

**Affiliations:** 1 Departments of Pediatrics and Cancer Biology, Lerner Research Institute and Cleveland Clinic, Cleveland, OH; 2 Department of Chemistry, Cleveland State University, Cleveland, OH; 3 Department of Inflammation and Immunity, Lerner Research Institute and Cleveland Clinic, Cleveland, OH; 4 Department of Pathology and Laboratory Medicine, Cleveland Clinic, Cleveland, Ohio, USA; 5 Division of Anatomic Pathology, Department of Pathology, University of Alabama at Birmingham, Birmingham, AL.

## Abstract

Barth syndrome is an X-linked syndrome characterized by cardiomyopathy, skeletal myopathy, and neutropenia. This life-threatening disorder results from loss-of-function mutations in *TAFAZZIN*, which encodes a phospholipid-lysophospholipid transacylase located in the mitochondria inner membrane. Decreased cardiolipin levels and increased monolysocardiolipin levels perturb mitochondrial function. However, the mechanism(s) leading to myopathies and neutropenia are unknown, and no currently effective therapy exists. To address these knowledge gaps, we generated *tafazzin*-deficient zebrafish. Neutropenia developed 5 days post-fertilization, but surprisingly no cardiac or skeletal myopathies were detected into adulthood. *tafazzin* mutants displayed multiple metabolic disturbances like those observed in humans with Barth syndrome. These include increased monolysocardiolipin: cardiolipin ratios, high levels of 3-methylglutaconic acid, decreased ATP production, increased levels of lactic acid, and hypoglycemia. There were also widespread effects on amino acid and unsaturated fatty acid synthesis. Despite these metabolic disturbances, zebrafish displayed a normal lifespan and fertility. Cardiolipin abnormalities were detected in both larvae and adult tissues, specifically in the heart and whole kidney marrow. Surprisingly, adult *tafazzin* mutants exhibited a higher number of neutrophils compared to wildtype fish. Further investigation revealed signs of inflammation as evidenced by elevated levels of *il6* in the whole kidney marrows and hearts of adult fish. Our comprehensive studies demonstrated that while mitochondrial dysfunction and metabolic defects were evident in *tafazzin*-deficient zebrafish, these disturbances did not significantly affect their development nor survival. These findings suggest that zebrafish may possess salvage pathways which compensate for Tafazzin loss or that humans have a unique vulnerability to the loss of TAFAZZIN.

Barth syndrome (BTHS; Online Mendelian Inheritance in Man #302060) is an X-linked inherited disorder due to mutations in *TAFAZZIN*. Patients with a pathogenic *TAFAZZIN* variant develop dilated cardiomyopathy, skeletal myopathy, and neutropenia. Death occurs during early childhood from septicemia and/or cardiac decompensation^1^. Additional clinical features include skeletal myopathy, exercise intolerance, delayed motor milestones, and learning disabilities. Metabolic disturbances produce growth delay, lactic acidosis, 3-methylglutaconic (3-MGC) aciduria, hypoglycemia, and low cholesterol ^2–6^. The Barth Syndrome Foundation estimates an incidence of 1 per 300,000–400,000 live births. The variability in clinical presentations of BTHS patients remains poorly understood ^7–10^.

*TAFAZZIN* contains 11 exons and is located on Xq28. The gene is highly conserved across species, including yeast, nematodes, zebrafish, frogs, and fruit flies, but exon 5 is specific for primates (Figure 1A)^3^. More than 120 different mutations throughout the *TAFAZZIN* genome have been identified. Most are missense or small indels, but a minority of patients have large exon or whole gene, deletions. Frameshift mutations resulting in a premature termination codon, and mutations affecting splice sites have also been identified^11^. No genotype/phenotype correlations have been identified, and marked phenotypic variation among males may occur within a family ^5,11^.

Tafazzin is required to remodel newly synthesized tetralineoyl-cardiolipin (CL), an essential component for mitochondrial function and structure. Cardiolipin has a vital role in the generation of mitochondrial cristae,^12^ the integrity of the inner mitochondrial membrane (IMM), and the stability of IMM-associated electron transport chain (ETC) proteins ^4,13^. Structural defects induced by *TAFAZZIN* mutations lead to metabolic disturbances such as reduced activity of respiratory chain complex proteins, Krebs cycle intermediates deficiency, and reduced ATP production. These abnormalities manifest themselves in high-energy demanding tissues such as muscle and heart ^14^.

CL metabolism is highly conserved and confined to the mitochondrial membrane.^11^ Three enzymatic pathways are involved in CL remodeling. As a transacylase on the outer face of IMM, TAFAZZIN transfers an acyl chain of phosphatidylethanolamine (PE) and/or phosphatidylcholine (PC) to monolysocardiolipin (MLCL), allowing for the formation of a fully mature form of CL. The lysocardiolipin acyltransferase (LCLAT1) and monolysocardiolipin acyltransferase 1 (MLCLAT1) are two other acyltransferases that reside, respectively, on the endoplasmic reticulum mitochondria-associated membranes and the IMM. Both LCLAT1 and MLCLAT1 use the acyl chain of acyl-CoA to ensure MLCL reacylation (Figure 1B). MLCLAT1 is a splice variant of the *HADHA* gene encoding for the α-subunit of the human trifunctional enzyme^15^. TAFAZZIN and MLCLAT1/HADHA resynthesize CL in mitochondria. ALCAT1 resynthesizes small fractions of MLCL to mature CL in the mitochondria associated membrane^16^. Enzyme expression varies between human tissues with the highest level of each gene found in heart and skeletal muscle (Figure 1C). *TAFAZZIN* mutations impair tetralinoleoyl-CL synthesis and increase MLCL, resulting in an increase of MLCL:CL ratio. This increased MLCL:CL ratio is the most sensitive and specific assay for the diagnosis of BTHS^14^. Still, little is known about the pathogenesis of myopathies and neutropenia.

Three major obstacles to understanding the pathophysiology of BTHS and developing more effective therapies are the rarity of the disease, limited availability of affected human tissues, and paucity of post-natal organismal models. Mouse models have been developed to study *Tafazzin* deficiency, producing variable phenotypes ^17–19^. Some strains of *Tafazzin* knockout mice exhibited severe cardiomyopathy, albeit delayed, characterized by impaired cardiac function, structural abnormalities, and increased mortality rates. These mice show significant alterations in heart morphology and function. In contrast, other *Tafazzin* knockout mouse strains maintain normal heart function, displaying no apparent signs of cardiomyopathy.^17^ These differences highlight the complexity of Tafazzin’s role in cardiac physiology and suggest that genetic background and environmental factors may influence the phenotypic outcomes of TAFAZZIN mutants.

Zebrafish offer a robust vertebrate model for studying heart, blood, and muscle development and disease^20–25^. Zebrafish harbor a single homologous *tafazzin* gene and 60% amino acid conservation between humans and fish (Figure 1A). Here, we report that *tafazzin* deficiency resulted in viable zebrafish with normal lifespan and without cardiomyopathy or skeletal myopathy. The fish were neutropenic at 5 days post fertilization (dpf) which corrected by adulthood. Metabolomic studies and RNA-Seq analysis demonstrated mitochondrial dysfunction and metabolomic differences between wild-type and *tafazzin*-deficient fish. Our studies demonstrated that while mitochondrial dysfunction and metabolic defects were present in *tafazzin*-deficient zebrafish, these disturbances were not severe enough to impair development and survival.

## MATERIALS AND METHODS

### Zebrafish husbandry.

Wild-type zebrafish were obtained from the Zebrafish International Research Center, ZIRC (https://zebrafish.org/). Animals were maintained according to standard protocols^26^. All experiments were approved by the Cleveland Clinic Institutional Animal Care and Use Committee. Fish were euthanized by immersion in ice water. This study is reported in accordance with ARRIVE guidelines (https://arriveguidelines.org). All methods were performed in accordance with the relevant guidelines and regulations.

### Mutagenesis of zebrafish tafazzin.

We designed gRNA to target exon 3 of *tafazzin* using the online Integrated DNA Technologies Alt-R^™^ CRISPR HDR Design Tool (**Supplemental Table 1**). We followed the IDT protocol Ribonucleoprotein delivery using the Alt-R^™^ CRISPR-Cas9 System to prepare samples for the zebrafish microinjections. The Alt-R CRISPR-Cas9 crRNA was synthesized by IDT and was duplexed with Alt-R CRISPR-Cas9 tracrRNA (IDT: 1073190) at 95°C for 5 minutes then allowed to cool to room temperature. Cas9 protein was purchased as Alt-R^®^ S.p. Cas9 Nuclease V3 (IDT: 1081058) and diluted to a concentration of 0.5 ug/ul. The gRNA was combined with Cas9 and incubated at 37°C for 10 minutes. One-cell stage embryos were injected with sgRNA and Cas9 into 1-cell–stage embryos. Genomic DNA was extracted at 1–2 days after injection for restriction site polymorphism-based genotyping and resulting alleles sequenced.

### Genotyping.

For genotyping, small DNA fragments were amplified using the primers listed in **Supplemental Table 2.** The *tafazzin* genotyping protocol is based on the elimination of the restriction enzyme site SmlI. The genotyping of the *tafazzin* allele was based on the 1 bp deletion. The PCR products were separated using 3.5% MetaPhor^™^ agarose gel (Lonza #50181).

### Chemical treatment.

Rotenone (Sigma) was dissolved in dimethyl sulfoxide (DMSO) to prepare a stock solution. The stock solution was diluted in egg water until reaching the working concentrations.

### RNA extraction and cDNA synthesis.

Groups of 25 larvae were collected at 5 days post-fertilization (dpf) and euthanized by immersion in an ice slurry. Each group represented an independent biological sample, and this process was performed in triplicate for each experiment. Adult fish were fasted for approximately 17 hours before dissection. Dissection surfaces and tools were cleaned and treated with RNAzap (Invitrogen^™^ #AM9780) prior to each dissection. For RNA extraction from the heart and whole kidney marrow, each *tafazzin* maternal zygotic and wild-type fish was dissected. Hearts were imaged prior to processing. Larvae pools, each individual heart and whole kidney marrow were placed in a 1.5 ml microcentrifuge tube with 500 μl TRIzol. Tissue was homogenized using a homogenizer (VWR Scientific), RNA isolated using TRIzol, and cDNA synthesized by iScript cDNA Synthesis Kit.

### Quantitative RT-PCR.

All assays were performed using Applied Biosystems PowerUp^™^ SYBR^™^ Green Master Mix (Thermo Fisher Scientific), Applied Biosystems MicroAmp Fast Optical 96-well 0.1 mL Reaction Plates (Thermo Fisher), and Applied Biosystems MicroAmp Optical Adhesive Film (Thermo Fisher) on Applied Biosystems QuantStudio 3 Real-Time PCR System (Thermo Fisher). For each reaction, 30ng total RNA was used. All assays consisted of three technical replicates and at least three biological replicates. Primers used are listed in **Supplemental Table 3.** The efficiency of each primer set was tested prior to experimental use following the equation: PCR efficiency (%) = (10^(−1/S)^ – 1)×100, where S=slope of the standardized curve. Expression of mRNA in mutants relative to wild-type was normalized to β-Actin and calculated by the Δ ΔC_T_ method.

### RNA-Seq.

RNA was extracted from pools of 25 larvae at 5 dpf using TRIzol reagent. Three pools of maternal zygotic *tafazzin* mutants and 3 pools of wild-type from the same clutch were compared. RNA quality was determined by Bioanalyzer (Agilent), and the *tafazzin* mRNA expression was measured by RT-qPCR. Sequencing reads generated from the Illumina platform were assessed for quality and trimmed for adapter sequences using TrimGalore! v0.4.2 (Babraham Bioinformatics), a wrapper script for FastQC and cutadapt. Reads that passed quality control were aligned to the zebrafish reference genome (GRCz11.112) using the STAR aligner v2.5.3. The aligned reads were analyzed for differential expression using Cufflinks v2.2.1, a RNASeq analysis package which reports the fragments per kilobase of exon per million fragments mapped (FPKM) for each gene using annotations from ZFin (v2024.08.19) annotation for GRCz11. Differential analysis was performed using the cuffdiff command in a pairwise manner for each group. Differential genes were identified using a significance cutoff of q-value<0.05(FDR < 5% using Benjamini Hochberg adjustment for multiple-testing correction). We performed a gene enrichment analysis for the genes under selection on each method using WebGestalt ^27^ and searching for Kyoto Encyclopedia of Genes and Genomes pathways (KEGG). Heatmaps were made using Heatmapper clustering web-based tool (http://heatmapper.ca) ^28^.

### Histology.

Fish were euthanized and fixed in 4% paraformaldehyde and decalcified using 5% trichloroacetic acid. Sectioning and staining were performed. To detect neutrophils, we fixed 5 dpf larvae for 2 hours at RT, rinsed three times with PBS for 10 min, and added Sudan black (380B-1KT; Sigma-Aldrich) for 20 min, then rinsed twice in 70% ethanol for 2 min and PBS three times. The larvae were bleached for depigmentation and imaging. All images were taken using ZEISS stereoscopes (Stemi 508 and Discovery V8) and an AxioImager M2 microscope with a camera (Axiocam). Neutrophils were counted for each larvae using the counter ImageJ plugin which allows the user to label each neutrophil that they click.

### Electron microscopy.

Larvae were collected at 5 dpf, anesthetized with tricaine (Syndel), and then fixed with fresh 4% formaldehyde in 1% glutaraldehyde in PBS at 4C overnight. The larvae were transferred to 1% osmium tetroxide and dehydrated using a graded ethanol series followed by treatment with propyleneoxide and embedded in Epon-812 resin. Ultra-thin sections (50–60 nm) were mounted on grids and post-stained with 3% uranylacetate in 50% ethanol and 1% lead citrate in 0.1 M sodium hydroxide and imaged under a Tecnai G2 Spirit BioTWIN is a 20–120 kV Transmission Electron Microscope (TEM) (Cleveland Clinic Imaging Core).

### CL and MLCL analysis.

Cardiolipins were extracted by a modified Folch method. A pool of 20 larvae at 5 dpf were homogenize in methanol (n=3 biological replicates). 10μL of CL (14:0)_4_ disodium salt (internal standard) at 0.778mM was added to homogenate followed by chloroform (1:1 v/v%). The mixture was shaken for 2 minutes, placed on ice for 15 minutes, then centrifuged at 10 °C for 5 minutes at 2000×g. The organic layer was transferred to a new tube and the aqueous layer was extracted again with chloroform/methanol (2:1, v/v%). Two organic layers were combined and centrifuged at 10 °C and 2000 × *g* for 5 min. The supernatant dried under N_2_ at room temperature. The residue was dissolved in 100 μL of the reconstitution solution and injected for UPLC-MS/MS (Shimadzu Nexera/ SCIEX Qtrap 5500) analysis.

The chromatographic separation was carried out with a mobile phase consisting of 0.1% ammonium hydroxide in acetonitrile/water (90:10, v/v%) at a flow rate of 0.4 mL/min on a XBridge^®^BEH C_18_ XP column (2.1 mm × 50 mm, 2.5 μm) (Waters/ Milford) with a 10.0 μL injection volume and the total run time 2 min per sample. Mass spectrometer was operated on multiple monitoring mode (MRM) to monitor the following transitions: 723.6 → 279.3 for CL (18:2)_4_, *m/z* 582.5→281.3 for the MLCL and *m/z* 619.7→ 227.3 for CL (14:0)_4._

### 3-MGC analysis.

Three biological replicates were prepared, each consisting of a pool of 20 larvae at 5 dpf. The larvae were homogenized in methanol, followed by the addition of an internal standard (tricarballylic acid 25 μL, 1mM). Samples were dried and 100 μl of 3N HCl-butanol was added. After 30 minutes at 65 °C, samples were dried and reconstituted in mobile phase (B). The reconstituted samples were injected for UHPLC-MS/MS analysis using an Xterra MS C18 column (2.1×50mm, 3.5μm, Waters/ Milford) with a Shimadzu Nexera UHPLC and SCIEX QTrap 5500 mass spectrometer. Mobile phases consisted of 0.1% formic acid in water (A) and acetonitrile (B). The gradient program as following: 0.5 min hold at 20 % (B), 70% (B) at 1.5 min hold for 3 minutes, 95 % (B) at 5 min, and 20% (B) at 8 min. Mass spectrometer was operated on multiple monitoring mode to monitor the following transitions: m/z 257→127 for 3-MGC and m/z 345→271for internal standard.

### Untargeted metabolomic analysis.

Three biological replicates were prepared, each consisting of a pool of 25 larvae at 5 dpf, which were homogenized with 250 μl cold methanol:acetic acid (95%:5% acetic acid) followed by addition of the internal standard (tricarballylic acid 25μL, 1 μM). The tubes containing each biological replicate were gently homogenized and then centrifuged. The supernatant was collected and dried under nitrogen. Samples were reconstituted in 100μL of methanol:water (v/v% 1:1). LC/QTOF analysis was conducted using an Agilent 6545 QTOF Mass Spectrometer coupled with an Agilent 1290 Infinity II UHPLC system. Chromatographic separation was achieved on a Waters Atlantis Premier HILIC-Z column (2.1mm × 150 mm, 1.7 μm). Analysis in negative mode was performed with the mobile phase consisting of (A) 10 mM ammonium acetate, 5 μM medronic acid in aqueous solution adjusted to pH 9 by ammonium hydroxide, and (B) acetonitrile. Analysis in positive mode have been performed with the mobile phase (A) 10 mM ammonium formate, 0.1% formic acid in aqueous solution, and (B) acetonitrile. The gradient elution profile for both ionization modes was as follows: 0–1 min, 90% B; 1–21 min, 70% B; 21–23 min, 40% B; 23–28 min, 10% B; 28–30 min, 10% B; 30–32 min, 90% B; 32–34 min, 90% B. Post-run equilibration time was set to 5 min, with a flow rate of 0.400 mL/min, a column temperature of 35 °C, and an injection volume of 3 μL.mRaw data were processed with Agilent MassHunter Profinder software (Version: B.10.0.2, https://www.agilent.com/en/product/software-informatics/mass-spectrometry-software/data-analysis/mass-profiler-professional-software/masshunter-profinder-mass-profiler-professional) for batch recursive feature extraction analysis. Metabolite’s annotation was performed with home library (retention time and MS/MS parameters) and with METLIN database (cut-off match 80%). Abundance of each metabolite was normalized to the internal standard (tricarballylic acid and to the number of larvae).

### Oxygen consumption rate (OCR).

OCR was measured using the XF96 Extracellular Flux Analyzer. The Seahorse XF analyzer (Seahorse Bioscience) assessed mitochondrial function by measuring the OCR in response to modulators of key components of the electron transport chain: (1) oligomycin, which inhibits ATP synthase (complex V) and decreases OCR linked to ATP production. (2) FCCP is an ionophore that collapses the proton gradient and disrupts the mitochondrial membrane potential by allowing protons to leak across the mitochondrial membrane. The FCCP-stimulated OCR can be used to calculate spare respiratory capacity, defined as the difference between maximal respiration and basal respiration. Spare respiratory capacity is a measure of the ability of the cell to respond to increased energy demand or under stress. (3) Rotenone (complex I)/Antimycin A (complex III). This combination shuts down mitochondrial respiration and allows the calculation of nonmitochondrial respiration driven by processes outside the mitochondria.

All drugs were dissolved in egg water and then loaded into XF96 extracellular flux assay plate (Seahorse Bioscience). Before successive injections of these three drugs, basal OCR (± TCS) was measured for approximately 1 hour (12 cycles, each consisting of 1.5 min mixing and 3 min measurement). Then, sequential injections of oligomycin, FCCP, and rotenone/antimycin-A with a final concentration of 12.5 μM, 2 μM 1 μM respectively were performed, and OCR measurements were recorded for 3 min. OCR measurements were recorded for 3 minutes (following 1.5 minutes of mixing) for 16, 7, and 10 times, respectively. Proton leak (which is defined as [lowest OCR measurement following oligomycin addition] - [OCR following rotenone/antimycin-A treatment]), ATP-linked respiration (which is defined as [final basal OCR measurement before oligomycin addition] - [lowest OCR measurement following oligomycin addition]), and spare respiratory capacity (which is defined as [maximal respiration following FCCP injection] - [final basal OCR measurement before oligomycin addition)] were calculated by the XF Cell Mito Stress Test Report Generator (Seahorse Bioscience) ^29,30^.

### ATP measurement.

Intracellular ATP levels were determined using a bioluminescence ATP determination kit (Invitrogen). Three independent pools of 5 larvae each were collected into separate 1.5 ml microcentrifuge tubes and homogenized in 90 μl of 2:2:1 mixture of acetonitrile:methanol:dH_2_O at −20°C. The resulting homogenate was centrifugated at 4°C at 15,000 rpm for 10 min, and the supernatant from each sample was isolated in fresh tube and stored at −20 °C. The assay kit was carried out according to the manufacturer’s instructions and emission read out was measured with a Gen5 plate reader (BioTek Instruments, St. Winooski, VT). A 50-fold dilution of the extracted larvae supernatant was made in dH2O, and 10 μl of the resulting solution was used per well for the ATP assay. Each condition was carried out in triplicate. Results were expressed as relative ATP levels.

### Cell preparation and flow cytometry.

Seven or eight fish of each genotype were fasted for approximately 17 hours before dissection. The SL and sex of each fish was recorded prior to dissection; surfaces and tools were cleaned prior to each dissection. Whole kidney marrow were placed in a 1.5 mL microcentrifuge tube on ice with 100 μl 4% fetal bovine serum (FBS) (Thermo #S10350H) in 1x PBS (Invitrogen) (v/v). The solution containing the organ was gently massaged through a 40 μm nylon cell strainer (Fisher #22363547) using the end of black rubber plunger from a 3 ml syringe and collected in a 50 mL conical tube. The filtered contents were washed with 3 mL of 4% FBS/PBS and pelleted by centrifugation for 5 minutes at 800 rpm. The supernatant was removed, and the step repeated. After the second centrifugation, the supernatant was removed, and the pellet resuspended in the residual fluid. This solution was then passed through the cell strainer cap of a 12 × 75 mm 5 mL round-bottom flow tube (Falcon). Eight or nine zebrafish kidneys were isolated under the stereoscope and mechanically dissociated using a 40-μm filter, rinsed in PBS-4%FBS, and centrifuged for 5 min at 800 g twice. The supernatant was aspirated, and the cells were resuspended in PBS-4% FBS. Cells were analyzed using a LSR Fortessa (BD Biosciences). The data were analyzed with FlowJo v10.5.3 software (FlowJo LLC, https://www.flowjo.com).

### Kidney touch preparation.

Fish of each genotype were fasted for approximately 17 hours before dissection. The standard length and sex of each fish was recorded prior to dissection; surfaces and tools were cleaned prior to each dissection. Kidneys were dissected from adult zebrafish, and gently touched to a clean glass slide, multiple times per slide. Slides were stained using Fisher HEMA 3. Manual counts of bone marrow cells were performed by a trained hematopathologist.

### Statistical analysis.

All statistical calculations were carried out with Prism 9.0 (GraphPad Software, https://www.graphpad.com). Parametric data are presented as Mean ± SEM. Each *n* value is indicated by a dot in the histogram, with each individual *n* value representing an individual animal. Statistical analysis used are unpaired two-tailed *t* tests or 1-way ANOVA with Tukey’s multiple comparisons test. Definition of statistical significance is considered P<0.05.

## RESULTS

### Generation and characterization of zebrafish mutants.

**FIGURE 1.**
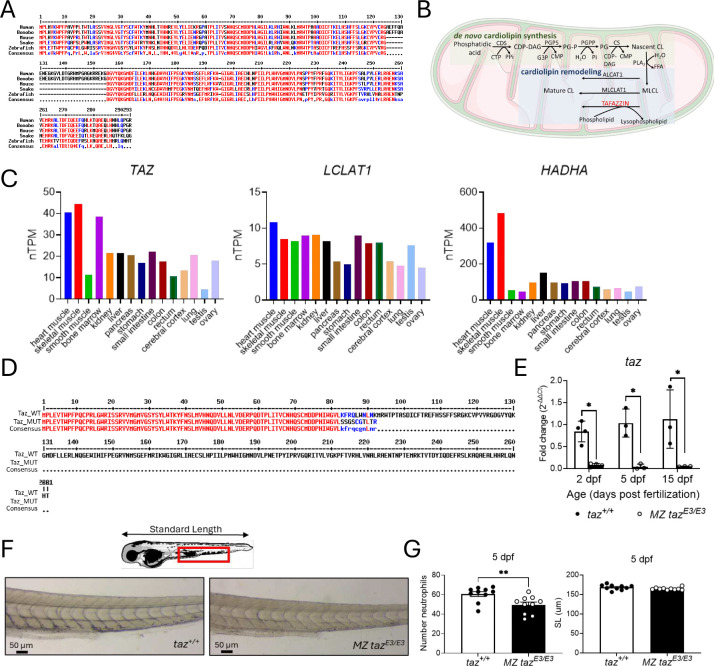
CRISPR/Cas9 genome editing of zebrafish *tafazzin*. A) Amino acid sequence alignment of TAFAZZIN in selected vertebrates. Conserved sequences indicated in red, similar in blue, and variable in black. Found only in primates, the *TAFAZZIN* gene contains an additional exon which encodes a unique 30 amino acid sequence. B) Cardiolipin synthesis and remodeling pathways. C) mRNA expression of enzymes (*HADHA*; Hydroxyacyl-CoA Dehydrogenase Trifunctional Multienzyme Complex Subunit Alpha; *LCLAT1*, Lysocardiolipin Acyltransferase 1; *TAZ*, TAFAZZIN) involved in cardiolipin remodeling in different human tissues (https://www.proteinatlas.org). D) Protein sequence alignment of wild-type Tafazzin and mutant (*taz*^*E3*^) E) RT-qPCR of *tafazzin* showing decreased transcript, perhaps secondary to nonsense-mediated decay, comparing to wildtype. F) Sudan black staining of larvae for neutrophils at 5 dpf G) Neutrophil quantitation and standard length at 5 dpf. *p<0.05; **p<0.01; MZ, maternal zygotic.

Using CRISPR/Cas9 editing, we targeted exon 3. The new mutation caused a 1 base pair deletion, resulting in a frameshift. This introduced a premature termination codon, leading to the production of a truncated protein. This *tafazzin* mutant allele was named *taz*^*lri113*^ here denoted as *taz*^*E3*^ (Figure 1D). Since zebrafish do not have sex chromosomes ^31^, we created homozygous *tafazzin* mutants. Unexpectedly, homozygous zygotic mutants (*taz*^*E3/E3*^) survived for at least two years without a visible phenotype. They were fertile and produce viable progeny. Since maternally derived factors may play an important role in early development and survival, we created maternal and zygotic *taz*^*E3/E3*^ mutants to investigate the effects of both a maternal and zygotic loss of Tafazzin. To create maternal zygotic (MZ) mutants, we incrossed the homozygous zygotic mutants (*taz*^*E3/E3*^) and compared the progeny with an incross of homozygous wildtype (*taz*^+/+^) siblings (**Figure S1**). Maternal zygotic *taz*^*E3/E3*^ larvae showed reduced *tafazzin* mRNA levels at 2, 5, and 15 dpf (Figure 1E). We tested three different Tafazzin antibodies by western blot to determine absence of Tafazzin protein, but none were able to detect the zebrafish protein.

Because BTHS is associated with cardiomyopathy, skeletal myopathy, and neutropenia, we checked heart and muscle morphology during the first 15 dpf but did not find any differences between wild-type and *taz*-deficient fish (data not shown). Next, we measured the number of neutrophils in zebrafish larvae at 5 dpf. A significantly lower number of neutrophils were detected in the MZ *taz*^*E3/E3*^ zebrafish larvae, with no differences in standard length detected (Figure 1F–G).

### Stress-induced responses in tafazzin-deficient larvae.

**FIGURE 2.**
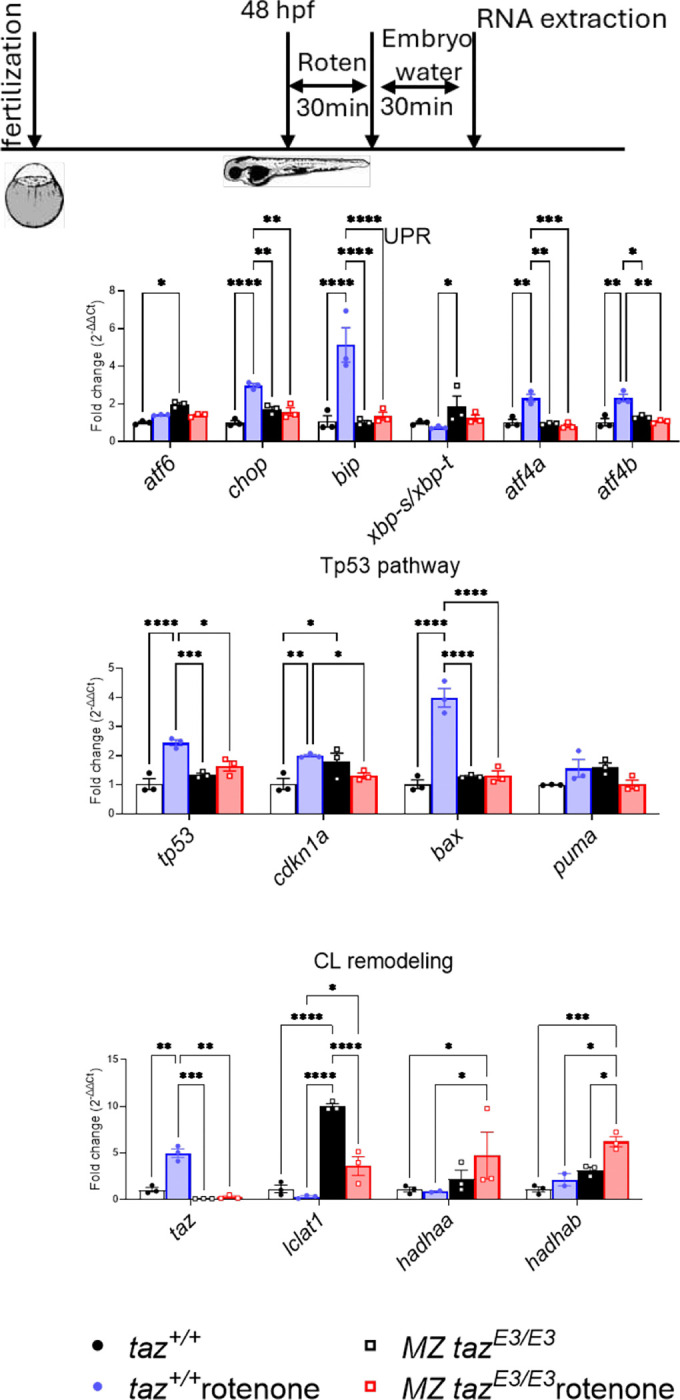
Mitochondrial stress marker analysis in tafazzin-deficient zebrafish: effects of rotenone inhibition of Complex I. *p<0.05; **p<0.01; ***p<0.001; **** p<0.0001.

To test how mitochondria respond to stress, we used rotenone, which inhibits complex I of the electron transport chain, leading to mitochondrial dysfunction. When we compared wildtype and *tafazzin*-deficient mutants, the mutants did not display any response to rotenone treatment. In contrast, wild-type fish showed activation of the unfolded protein response and Tp53 pathways, indicating that *tafazzin* mutants may have an impaired ability to respond to mitochondrial stress. (Figure 2).

### Developmental metabolic abnormalities in tafazzin mutants.

**FIGURE 3.**
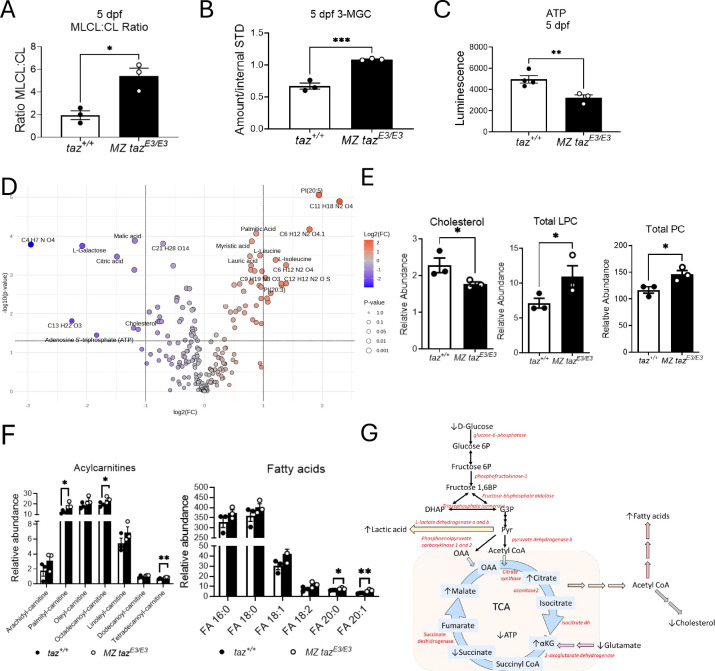
Metabolomic comparison of wild-type versus *tafazzin*-deficient 5 dpf larvae metabolomics. A) Total MLCL:CL ratio and B) 3-MGC C) ATP production. D) Untargeted metabolomics: volcano plot showing metabolites significantly upregulated (red) or downregulated (blue) in mutants compared to wildtype. The significant features highlighted in the volcano plot were defined as having a false discovery rate <0.05 and fold change >1.5. Blue color indicates MZ *taz*^*E3/E3*^ express lower levels on identified metabolites. Unique molecular formulas were determined by high-resolution, accurate intact mass, isotopic patterns, and MS/MS analyses. E) Cholesterol, Total LPC and Total PC E) Acylcarnitine and Fatty acids. G) Metabolic pathways affected in tafazzin-deficient fish are illustrated, with altered metabolites marked by arrows and enzymes with upregulated mRNA expression highlighted in red font. *p<0.05; **p<0.01; ***p<0.001.

The primary diagnostic metabolic measurements in BTHS are an increased ratio of MLCL:CL and elevated 3-methlyglutaconic acid (3-MGC) levels. We analyzed these parameters in the zebrafish MZ *taz*^*E3/E3*^ mutants and wildtype at 5 dpf. The rapid development of zebrafish ensures that all major organ systems, including the liver, are fully established by 5 dpf, a developmental stage comparable to full-term gestation in humans ^32^. Whole larvae extracts at 5 dpf displayed a significant increased ratio of MLCL:CL (Figure 3A) and elevated levels of 3-MGC acid (Figure 3B). ATP levels were significantly decreased in 5 dpf larvae (Figure 3C). Comparison of metabolic profiles between MZ *taz*^*E3/E3*^ and *taz*^+/+^ larvae at 5 dpf revealed approximately 69 metabolites significantly altered in the MZ *taz*^*E3/E3*^ mutant, highlighting the important role of *tafazzin* in zebrafish metabolism (Figure 3D–F, **Figure S2A-B**). Metabolic changes in the mutant included increased lactic acid and L-glutamic acid production and decreased levels of D-glucose, glutathione, and tricarboxylic acid cycle metabolites such as citric acid, malic acid, fumaric acid, and succinic acid. Increased lactate production, energy deficiency, abnormal tricarboxylic acid intermediates profiles and decreased fatty acid oxidation with concomitant accumulation of fatty acids’ catabolic lipotoxic intermediates are reported and in agreement with other BTHS models and human studies ^33–36^.

Since some BTHS patients exhibit mild hypocholesterolemia, we investigated the levels of cholesterol, phosphatidylcholine (PC) and lysophosphatidylcholine (LPC). PCs are generally the most abundant phospholipid class in cells and play a crucial role in facilitating the incorporation and distribution of cholesterol within cell membranes ^37^. LPC is primarily produced from the turnover of PC through the action of phospholipase A2 (PLA_2_). LPC can modulate lipid signaling pathways and membrane dynamics, further impacting cholesterol levels and its distribution in cells. Changes in LPC and PC levels can disrupt these processes, leading to alterations in cholesterol metabolism and distribution ^38^. We detected lower cholesterol levels in *taffazin* mutants, similar to those observed in BTHS patients, along with increased levels of LPC and PC (Figure 3E).

ATP can be produced through FA β-oxidation in mitochondria. Additionally, long-chain acylcarnitines (LCACs) are intermediate forms of FA transport, essential for delivering FAs from the cytosol into mitochondria. Consistent with a reduced TCA cycle and ATP production, we observed significantly higher levels of LCACs and FAs in the mutant zebrafish larvae (Figure 3F)^39^. These findings indicate attenuated and incomplete FA oxidation and were consistent with the metabolic profile observed in BTHS patients (Figure 3G).^36,40^

**FIGURE 4.**
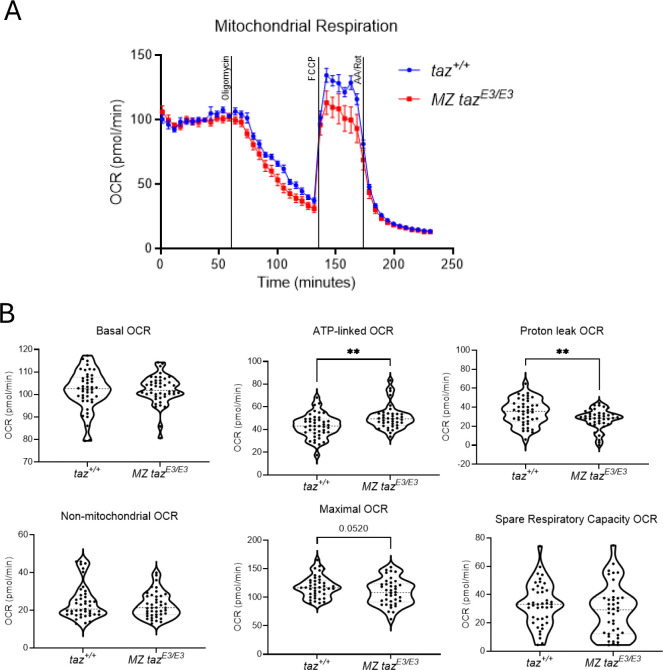
Mitochondrial dysfunction in *tafazzin*-deficient embryos. A) Diagram showing oxygen consumption rates (OCR) for wildtype (blue line) and MZ *taz*^*E3/E3*^ (red line) zebrafish embryo following the addition of oligomycin, FCCP, and rotenone. Error bars represent the standard error of the mean (SEM). B) Oxygen consumption rates for basal, non-mitochondrial respiration, maximal, proton leak, ATP-linked respiration and spare respiratory capacity. Each dot in the figure represents the value of one independent embryo. Results showing three independent experiments. Each experiment included 15 embryos per genotype. *p<0.05; **p<0.01.

The primary cause of BTHS is a disruption in the mitochondrial membrane phospholipid composition that affects mitochondrial bioenergetics and dynamics ^41^. We used embryos at 1 dpf to investigate how early mitochondrial dysfunction occurs by measuring key components of the electron transport chain using the Seahorse XF analyzer. Treatment with oligomycin, an inhibitor of ATP synthase (complex V), resulted in a decrease in OCR linked to ATP production. Additionally, FCCP was used to collapse the proton gradient and disrupt ATP synthesis (Figure 4A). Our results demonstrated that maternal zygotic *tafazzin* mutants exhibited significant defects in mitochondrial respiration compared to wild-type, characterized by decreased OCR, reduced proton leak respiration, and TAFAZZIN lower maximal respiratory capacity, despite an increase in oxygen consumption for ATP production and unchanged spare respiratory capacity (Figure 4A–B).

### Changes in the transcriptomic profile of tafazzin deficient zebrafish larvae.

**FIGURE 5.**
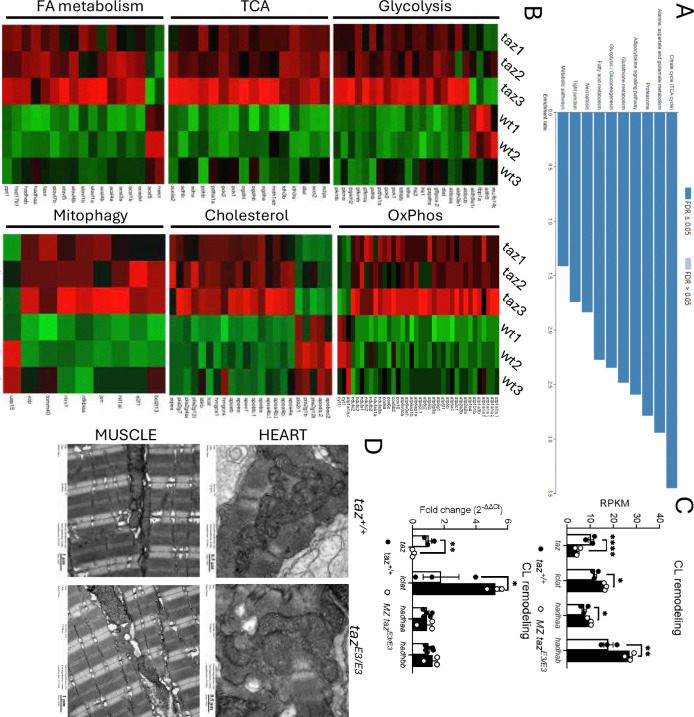
Changes in the transcriptomic profiling of *tafazzin*-deficient zebrafish larvae. A) KEGG enrichment analysis of DEGs. B) List of the DEGs. C) Reads Per Kilobase per Million mapped fragments (RFKM) of genes related to CL maturation and q values and validation by RT-qPCR. D) Electron micrographs show in the wildtype and *tafazzin* at 5 dpf—heart and muscle the presence of abundant mitochondria with double membrane system and cristae. There are no significant differences between the wildtype and *tafazzin* mutants—in the density and morphology of mitochondria. Likewise, the sarcomeres, well demarcated by the Z lines, show no significant differences between both groups in heart and muscle. Note the different scale between both tissue types micrographs.*p<0.05; **p<0.01.

We investigated the effects of *tafazzin* deficiency at the transcriptome level in 5 dpf larvae by RNA-seq (**Figure S3A-B**). Of the 4441 genes differentially expressed (DEG), 1700 genes were upregulated and 2741 were downregulated. The top 20 most upregulated and downregulated genes are listed in **in Supplemental Table 4**. Next, we performed functional analysis of differentially expressed genes using gene ontology (GO) analysis and gene set enrichment. Consistent with our metabolomic findings, transcriptomic analysis revealed significant alterations in the TCA cycle, glycolysis, and fatty acid metabolism pathways in tafazzin-deficient larvae. To validate the RNA-seq results, we analyzed specific genes involved in the TCA and glycolysis pathways by RT-qPCR (**Figure S3C**). These results collectively indicate that tafazzin deficiency leads to pronounced metabolic dysfunction (Figure 5A–B). Tafazzin deficiency is associated with mitochondrial dysfunction, potentially impacting oxidative phosphorylation and mitophagy. To investigate these pathways, we analyzed markers in our RNA sequencing results. Notably, genes involved in mitochondrial oxidative phosphorylation and mitophagy were significantly upregulated in *tafazzin*-deficient larvae (Figure 5B), suggesting a compensatory response to mitochondrial dysfunction (Figure 3G). This aligns with previous studies indicating that *tafazzin* mutations can lead to increased mitochondrial superoxide anions and alterations in mitophagy processes.^4,42^

Next, we analyzed the transcriptional profile of genes encoding enzymes involved in cardiolipin remodeling (*tafazzin, lclat, hadhaa,* and *hadhab)*. Due to an additional round of whole genome duplication during vertebrate evolution in teleost fish, zebrafish possess two paralogs of many important genes, including *HADHA* ^43^. As previously observed by RT-qPCR, *tafazzin* mRNA levels were decreased in the *tafazzin* mutants. Of note, we discovered a significant increase of *lclat* mRNA levels in mutants in both RNA-seq and RT-qPCR studies (Figure 5C).

### No changes in mitochondrial morphology in tafazzin-deficient larvae.

Despite the mitochondrial and metabolic defects observed in *tafazzin* mutants, transmission electron microscopy (TEM) did not reveal significant changes in mitochondrial morphology or number in 5 dpf larvae (Figure 5D). This lack of observed morphological changes, despite metabolic abnormalities, may suggest that mitochondrial dysfunction does not lead to detectable structural alterations.

### taz^E3/E3^ zebrafish survive until adulthood and display abnormal blood cell lineages in whole kidney marrow.

Since we did not observe any cardiac dysfunction in MZ *taz*^*E3/E3*^ mutant larvae, we investigated the effects of *tafazzin* deficiency in adult fish. No differences in heart size, standard length, survival, or fertility were noted between wild-type and MZ *tafazzin* mutants (**Figure S4A-D**). Histological staining with H&E did not reveal any differences in internal organs in one year old fish, including the heart, liver, kidney, and muscle. (**Figure S4E**).

**FIGURE 6.**
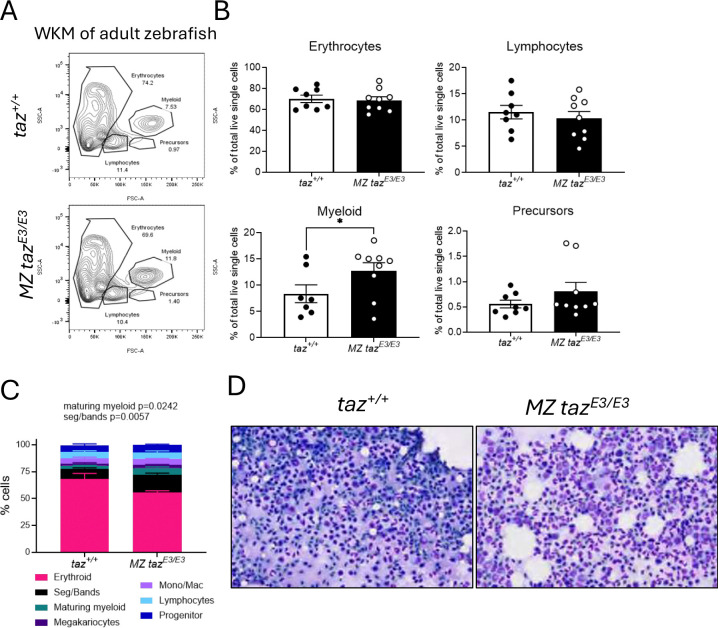
Adult *tafazzin*-deficient zebrafish exhibited increased MLCL:CL ratio in heart and whole kidney marrow but survive until adulthood. A) Flow cytometric analysis and B) quantification of blood cells. C) Differential cell counts in kidney marrows and D) kidney touch preparations of wildtype and *tafazzin* mutants at 60X magnification *p<0.05.

Given our earlier observation of reduced neutrophil numbers at 5 dpf, we analyzed the total cellularity of the whole kidney marrow to examine the different blood cell populations in adult fish. The kidney marrow serves as the site for definitive hematopoiesis in adult zebrafish, similar to the mammalian bone marrow ^24^. Surprisingly, we found a significant increase in the myeloid group in the whole kidney marrow by flow cytometry (Figure 6A–B). Furthermore, kidney touch preparation from adult fish showed significantly increased neutrophils in the *MZ taz*^*E3/E3*^ mutants (Figure 6C–D). These results, coupled with the result of neutropenia at 5 dpf in MZ *taz*^*E3/E3*^ larvae, suggest that blood cells may be more sensitive to loss of *tafazzin* in the zebrafish.

**FIGURE 7.**
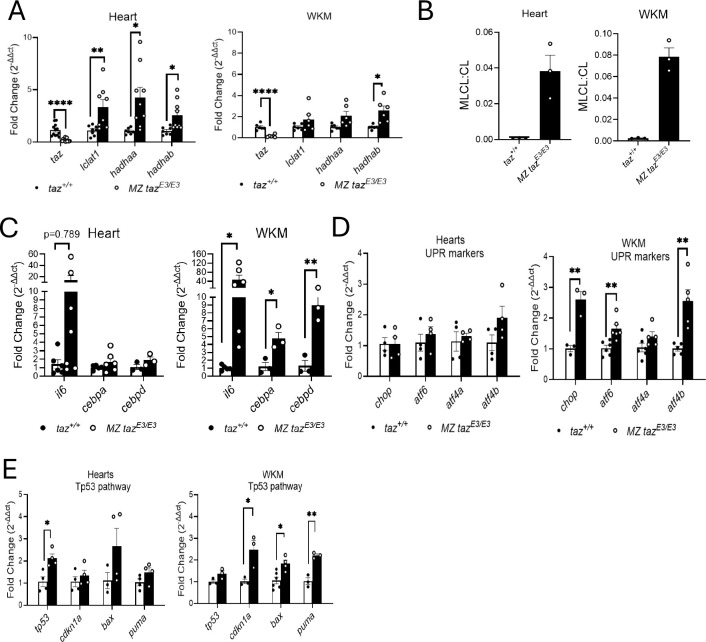
Inflammatory response in adult *tafazzin*-deficient fish. A) mRNA levels of CL remodeling enzymes in heart and whole kidney marrow (WKM). B) MLCL:CL ratio is reduced in heart and WKM. RT-qPCR results for C) Inflammation D) UPR, and E) Tp53 pathway markers. *p<0.05; **p<0.01; ***p<0.001; **** p<0.0001.

To investigate transcriptional differences in the internal organs, we extracted whole kidney marrow and hearts from adult zebrafish. As expected, we observed reduced expression of *tafazzin* mRNA in both tissues in *taz*^*E3/E3*^ comparing to wildtype. The mRNA levels for enzymes involved in cardiolipin remodeling were increased in both tissues, suggesting a compensatory mechanism for the deficiency in Tafazzin (Figure 7A). However, the MLCL:CL ratio in both tissues was increased in *tafazzin*-deficient zebrafish compared to wild-type, similarly to what it is observed in BTHS patients and other BTHS models (Figure 7B).

The mild neutrophilia in whole kidney marrow prompted us to evaluate proinflammatory markers using RT-qPCR in heart and whole kidney marrow. Interestingly, the cytokine *il6* was strongly upregulated in both tissues, while *cebpα* and *cebpδ*, were upregulated exclusively in whole kidney marrow, suggesting that the inflammatory response is more acute in the kidney (Figure 7C). This differential expression pattern may indicate that while the *tafazzin* deficiency triggers a widespread inflammatory reaction, the whole kidney marrow may be experiencing a more intense local response compared to other tissues. Furthermore, we examined UPR and TP53 critical stress pathways. Our results showed that UPR and TP53 activity were significantly elevated in the whole kidney marrow but not in the heart (Figure 7D–E). This finding aligns with the observed increase in inflammation in the kidneys.

## DISCUSSION

We report here a new organismal model for the study of BTHS that suggests a salvage pathway to rescue mitochondrial dysfunction and prevent development of cardiac and skeletal myopathies. To understand what happens with a global loss of *tafazzin* throughout development and characterize more completely the pathogenesis of BTHS, we used CRISPR/Cas9 gene editing to create *tafazzin*-deficient zebrafish. These fish exhibited neutropenia in early stages of development. Surprisingly, they did not develop cardiomyopathy or skeletal myopathy. Our zebrafish model reproduces multiple metabolic properties of BTHS: high 3-methylglutaconic acid, high MLCL:CL ratio, abnormal TCA cycle intermediates- associated with mitochondrial abnormality, ATP deficiency, hypoglycemia, and lactic acidosis.

For a rare disease like BTHS, organismal models are of great value ^44^. To date, there has been only one report of targeting *tafazzin* in zebrafish ^45^. Using antisense morpholinos, the Strauss lab knocked down *tafazzin* during early embryonic development and observed profound effects at 51 hours post fertilization: severe developmental and growth retardation, marked bradycardia and pericardial effusion, edema, and lethality. However, the effects of morpholinos include unusually severe phenotype, aberrant activation of tp53 pathways and a transient period of knockdown in the earliest stages of development^46^.

One possible reason for the discrepancy between the morpholino-induced phenotype and the phenotype observed in the CRISPR/Cas9-induced mutant may be genetic compensation. Phenotypic differences between knockdowns and genetic mutants are often observed in zebrafish, as well as mouse ^47^. One reason contributing to this phenomenon is the upregulation of genes and proteins in the genetic mutant background that compensates for the genetic loss of function ^48,49^. Often, phenotypic differences between knockdown and genetic mutants are most evident when the genetic mutant allele displays a high level of nonsense mediated decay (NMD), indicating that the NMD may in part trigger the compensatory response ^47,50,51^. Intriguingly, the *tafazzin*-deficient zebrafish created display a very high level of NMD, as the *tafazzin* transcript level in mutants is well below twenty percent of wildtype levels. This suggests that a compensatory response may be responsible for the milder than expected phenotype in the CRISPR/Cas9-induced *tafazzin*-deficient zebrafish.

Compensatory mechanisms likely contribute to the clinical variability observed in BTHS patients as well and can influence disease progression as well as severity ^6^. While the typical clinical phenotype includes cardiomyopathy, cyclic neutropenia, skeletal myopathy, and growth deficiency, BTHS may be underdiagnosed due to its variable spectrum of clinical presentations ^2,5,9,52–54^. As with the variability seen in cardiac manifestations, neutropenia in BTHS exhibits a range even among patients or family members with identical *TAFAZZIN* gene mutations ^2,5,6,52^. Neutropenia in patients with BTHS can be chronic, intermittent, cyclic, or absent. As the understanding of the clinical spectrum of BTHS grows, reports of BTHS diagnoses in adulthood have become more frequent ^9,10^.

The *tafazzin*-deficient fish created showed biochemical and genetic abnormalities associated with the loss of TAFAZZIN in humans. Mitochondrial dysfunction occurred as early as 1 dpf, as demonstrated by changes in oxygen consumption rate and proton efflux rate or extracellular acidification rate and gene expression patterns. While alterations in the MLCL:CL ratio was consistent with BTHS, there was no evidence of swollen or dysmorphic mitochondria on electron microscopy. The *tafazzin*-deficient fish may have three key compensatory responses to *tafazzin* deficiency: (1) metabolic adaptations, including increased TCA cycle activity and glycolysis to produce energy despite mitochondrial dysfunction; (2) enhanced oxidative phosphorylation and antioxidant production to manage oxidative stress; and (3) upregulated mitophagy to remove dysfunctional mitochondria and maintain cellular health. These compensatory mechanisms may ensure that the zebrafish can sustain essential physiological processes, support growth and development, and ultimately reach adulthood despite the genetic deficiency.

The possibility of a compensatory mechanism(s) acting downstream from cardiolipin in a *Tafazzin*-deficient background is supported by mouse studies^18^. Wang *et al* created eight mouse strains, each harboring the same genetic lesion. Great phenotypic variability was observed between strains, with some developing severe cardiomyopathy and early mortality while other maintained normal heart function and survival rates. Interestingly, all strains exhibited similar cardiolipin abnormalities. In mice strains permissive to *Taz* deficiency, those that exhibited normal heart function and preserved mitochondrial morphology, mitophagy markers were increased ^18^. Intriguingly, mitophagy makers were elevated in the *tafazzin*-deficient fish, indicating that a genetic compensatory mechanism acting downstream of cardiolipin may impact the phenotypic severity of *tafazzin* deficiency.

Uncovering the mechanism(s) involved in this process may shed new light on the pathophysiology of BTHS and aid in the identification of new treatment therapies. Two of the greatest obstacles to understanding the pathophysiology of BTHS and developing more effective therapies are the rarity of the disease and absence of an organismal model. Genetic ablation of Tafazzin in mice results in embryonic lethality. Zebrafish provide an excellent vertebrate model for studying heart, blood, and muscle development and disease. In creating a zebrafish line targeting exon 3 of tafazzin, we found that these mutants phenocopy the principal biochemical characteristics. The absence of myopathy suggests an alternative bioenergetic pathway.

The mild neutrophilia found in whole kidney marrow from otherwise healthy adult *tafazzin*-deficient fish may suggest underlying metabolic alterations. One potential mechanism involves the role of autophagy ^55^, a cellular process that degrades and recycles cellular components. During periods of metabolic stress, autophagy can release free fatty acids, which are then used in mitochondrial respiration to generate energy. This process may be particularly important in promoting neutrophil production, as these cells require substantial energy to perform their immune functions. Furthermore, the elevation of pro-inflammatory cytokines, such as Il6, observed at the mRNA level in *tafazzin*-deficient zebrafish suggests a state of chronic inflammation ^56^. The sensitivity of neutrophils to Tafazzin deficiency might be due to their high metabolic demands and reliance on mitochondrial function, which is compromised in the absence of functional Tafazzin. This could lead to altered energy metabolism and an increased need for neutrophil production as a compensatory mechanism. The increased neutrophil count could serve as an indicator of the systemic metabolic stress and chronic inflammatory state induced by Tafazzin deficiency, potentially providing insights into the broader pathophysiological consequences of impaired mitochondrial function.

Overall, our study provides a comprehensive analysis of mitochondrial dysfunction, metabolic abnormalities, and compensatory responses in *tafazzin*-deficient zebrafish. The observed metabolic alterations and compensatory responses align with clinical features of BTHS, and mouse models and underscore the complex interplay between mitochondrial function, metabolic regulation, and inflammation. These findings enhance our understanding of BTHS pathology and offer insights into potential therapeutic targets for managing mitochondrial disorders.

## Data Availability

RNA-Seq data are available in the GEO database (GEO GSE283499). All other data are available upon reasonable request to the corresponding authors.
